# Heterogeneous distribution of *Plasmodium falciparum *drug resistance haplotypes in subsets of the host population

**DOI:** 10.1186/1475-2875-7-78

**Published:** 2008-05-06

**Authors:** Sonja Schoepflin, Jutta Marfurt, Mary Goroti, Moses Baisor, Ivo Mueller, Ingrid Felger

**Affiliations:** 1 Swiss Tropical Institute, Department of Medical Parasitology and Infection Biology, Socinstr.57, 4002 Basel, Switzerland; 2 Papua New Guinea Institute of Medical Research, Madang, P.O. Box 378, M.P. 511, Papua New Guinea

## Abstract

**Background:**

The emergence of drug resistance is a major problem in malaria control. For mathematical modelling of the transmission and spread of drug resistance the determinant parameters need to be identified and measured. The underlying hypothesis is that mutations associated with drug resistance incur fitness costs to the parasite in absence of drug pressure. The distribution of drug resistance haplotypes in different subsets of the host population was investigated. In particular newly acquired haplotypes after radical cure were characterized and compared to haplotypes from persistent infections.

**Methods:**

Mutations associated with antimalarial drug resistance were analysed in parasites from children, adults, and new infections occurring after treatment. Twenty-five known single nucleotide polymorphisms from four *Plasmodium falciparum *genes associated with drug resistance were genotyped by DNA chip technology.

**Results:**

Haplotypes were found to differ between subsets of the host population. A seven-fold mutated haplotype was significantly reduced in adults compared to children and new infections, whereas parasites harbouring fewer mutations were more frequent in adults.

**Conclusion:**

The reduced frequency of highly mutated parasites in chronic infections in adults is likely a result of fitness costs of drug resistance that increases with number of mutations and is responsible for reduced survival of mutant parasites.

## Background

The emergence of drug resistance poses a major problem for malaria control. Research has focused mainly on elucidating the mode of action of antimalarial drugs and on the molecular mechanisms leading to drug resistance. The development of drug resistance mostly involves single nucleotide polymorphisms (SNPs) in genes encoding the drug targets, such as metabolic enzymes or transmembrane transporters. Molecular studies have identified a number of SNPs in the *Plasmodium falciparum *multi-drug resistance gene 1 (*pfmdr1*), the chloroquine resistance transporter (*pfcrt*), the dihydrofolate reductase (*pfdhfr*) and the dihydropteroate synthase (*pfdhps*) that were associated with drug resistance against the most commonly used antimalarials chloroquine (CQ) and the combination of antifolate sulphadoxine/pyrimethamine (SP). Allelic exchange experiments did provide direct evidence for the role of *pfdhfr *in resistance to pyrimethamine [1], *pfdhps *in resistance to sulphadoxine [2] and the role of *pfcrt *in resistance to CQ [3]. Reed *et al *[4] could show that mutations in *pfmdr1 *are not essential, but can modulate the level of drug resistance against CQ. Evidence from molecular epidemiological field studies confirmed associations of the mutations K76T [5-7] and N86Y [8-10] with *in vivo *drug resistance against CQ, whereas different combinations of mutations in *dhfr *and *dhps *were associated with *in vivo *drug resistance against SP [for example [11-14]].

A number of mathematical models have been designed to predict the transmission and spread of drug resistance [15-20]. Questions addressed include the role of transmission intensity on the spread of drug resistance or the possibility of a preferential transmission of resistant versus sensitive parasites. The effect of transmission intensity on the spread of drug resistance was discussed controversially [19,21]. A more recent review by Hastings & Watkins [15] proposed that transmission intensity does not have a direct impact on the evolution of drug resistance, but directly determines the dynamics of resistance via "effectors", such as intra-host competition, level of drug use in the population, extent of sexual recombination, proportion of malaria infections treated, or number of parasites in a human host.

Different authors studied the preferential transmission of resistant versus sensitive parasites. It has been proposed that moderately mutated parasites that result in high levels of parasitological failures following SP treatment, have a greater transmission potential than highly mutated parasites, because the latter are more likely to cause clinical failure within a short time after treatment and are therefore more often subject to rescue treatment [22]. A number of field studies have reported increased gametocyte carriage in blood of CQ or SP treated individuals [23-27] or in individuals carrying drug resistant strains [23,25]. However, these findings have been questioned by lacking evidence that parasites post SP-treatment fully develop to infective stages in the mosquito [28].

The field study presented here was conducted in Papua New Guinea (PNG) and investigated the distribution of drug resistance genotypes in subsets of the host population. In particular, the actually transmitted genotypes were characterized. This was possible by following individuals after radical cure treatment. The first *P. falciparum *positive blood samples of these individuals were genotyped for all known markers for drug resistance.

The underlying hypothesis of this study was that new infections following radical cure are not yet subject to major selective constraints, because these newly arriving infections do not have to compete against already persisting infections. Here all transmitted genotypes irrespective of their fitness are expected to be found. In contrast, parasites in asymptomatic chronic infections from community samples have been subject to within-host competition and differential fitness is likely to affect their long-term survival in the host. Asymptomatic individuals are therefore expected to carry a lower prevalence of mutated genotypes as a result of decreasing fitness with increasing number of mutations.

By molecular epidemiological studies in Madang, PNG, detailed information has been provided on the currently high levels of mutation rates in *Pfcrt *K76T (97%), *Pfmdr1 *N86Y (96%) and *Pfdhfr *S108N (82%) and C59R (74%)[29]. Another study in PNG observed a significant increase in the *dhfr *double mutant C59R + S108N over a period of two years (83% to 96%). This study was performed in 2002 and 2003, a few years after the introduction of SP as first line treatment in combination with 4-aminoquinolines [30]. They also found an increase in the quadruple mutant *dhfr *C59R + S108N + *dhps *A437G + K540E from 0 % to 8.2% and even though this was not significant, these results suggest that resistance to SP is rapidly developing in PNG. The genotype most frequently found in treatment failures contained a quadruple mutant in *pfcrt *(K76T + N326D + I356L + A220S) in combination with the *pfmdr1 *mutant N86Y and the double *pfdhfr *mutant S108N+C59R.

For the present investigation, which aims primarily at multi-loci haplotypes, the PNG field site provides optimal conditions. A haplotype, which is here defined as the genetic make up of an individual parasite clone at 25 loci from four marker genes of drug resistance, can be directly deduced after genotyping a single clone infection. As multiclonal infections are rare in PNG, with a mean multiplicity of infection (MOI) between 1.3 and 1.8 [31-33], the high prevalence of single-clone infections greatly facilitated the study of drug resistance haplotypes.

## Materials and methods

The present study made use of two sets of blood samples: (i) cross-sectional surveys including Papua New Guinean individuals of all ages, and (ii) first *P. falciparum *positive samples from a follow up after radical cure with artesunate in five to 14 year-old children. Both sets of samples were collected in the same villages in PNG, in the years 2003 – 2005.

In the treatment to reinfection study (TRS) in 2004/2005 [for details see [34]] a total of 206 children from five to 14 years of age were enrolled at the Mugil and Megiar elementary schools which are situated about 50 km north of Madang town, PNG. After obtaining written informed consent from the parents or guardians each child was clinically examined, two blood slides (thick and thin films) were prepared for microscopical determination of malaria infections and a venous blood sample was collected at baseline. Subsequently, all children were treated with a seven-day course of artesunate monotherapy according to PNG national treatment guidelines (i.e. 4 mg/kg at day 1, 2 mg at days 2–7). After treatment, two-weekly active follow ups were conducted at the schools to check for new malaria infections and presence of febrile illness. Therefore, each child was clinically assessed, a rapid diagnostic test (RDT) (ICT Diagnostics, South Africa) was performed, blood slides were prepared and 250 μl of blood were collected by finger prick every two weeks.

Positive samples were identified by microscopy and LDR-FMA [35] as described in more detail in Michon *et al *[34]. All baseline and first PCR positive samples after treatment were genotyped for the highly polymorphic marker gene merozoite surface protein 2 (*msp2*) and compared by PCR-RFLP [36,37] in order to distinguish new from recrudescent infections. Samples that were typed as recrudescent infection, but were collected as late as 10 weeks or more after the baseline survey, were additionally genotyped for a second marker gene (*msp1*) [38], because recrudescence seemed to be unlikely after an interval of this length.

In the same villages two household-based cross sectional surveys were conducted in 2003 and 2004 which included participants of all age groups. Upon receiving informed consent a questionnaire was completed for each participant, blood slides prepared for microscopical examination and a venous blood samples was collected for further laboratory analysis. All samples were *msp2*-genotyped using PCR-RFLP as described above to determine the multiplicity of infection (MOI).

All samples that were determined to be single or double infections by *msp2 *genotyping were further analysed for mutations in drug resistance genes by DNA chip technology [39]. This method allows parallel identification of 25 single nucleotide polymorphisms (SNPs) that were found to be associated with drug resistance against a number of different antimalarial drugs. It is based on PCR amplification of target sequences within the genes *Pfmdr1*, *Pfcrt*, *Pfdhfr *and *Pfdhps*. A primer extension reaction with fluorescent labelled ddNTPs follows this PCR step. The extended primers are subsequently hybridized on a microarray carrying the antisense DNA of the extension primers and scanned at different wavelength using an Axon 4100A fluorescent scanner to determine the incorporated ddNTP. Pictures were acquired and analysed using the Axon GenePix^® ^Pro (version 6.0) software. The codons investigated with this method include N86Y, Y184F, S1034C, N1042D and D1246Y in *Pfmdr1*, the codons K76T, H97Q, T152A, S163R, A220S, Q271E, N326D/S, I356L/T and R371I in *Pfcrt*, the codons A16V, N51I, C59R, S108N/T and I164L in *Pfdhfr *and the codons S436A, A437G, K540E, A581G, I640F and H645P in *Pfdhps*.

The dataset for statistical analysis consisted of all cross-sectional samples that had been genotyped as single-clone infections and all new infections (irrespective of malaria symptoms) plus baseline samples with MOI = 1 from TRS study. Samples with MOI = 2 were taken into consideration, if it was possible to unequivocally determine the haplotype of these samples, i.e. samples showing a mixed infection for more than two loci were not included. Samples from the cross sectional surveys were grouped into adults (> 14 years of age) and children five -14 years of age. All individuals from the household surveys who had received any antimalarial treatment during the last two months prior to the survey were excluded.

For simplified presentation of haplotypes, only SNPs of which the mutated alleles were actually detected in this study area are itemized in the haplotype descriptions, whereas SNPs found only in the wild type form are not listed. Thus, the presented haplotype provides molecular typing information on alleles at the following 11 positions: *dhfr*59, *dhfr*108, *dhps*437, *dhps*540, *mdr*86, *mdr*184, *mdr*1042, *crt*76, *crt*220, *crt*326 and *crt*356. Only samples with a complete set of these 11 polymorphic SNPs were taken into consideration.

To compare the haplotype frequencies between datasets logistic regression statistics was applied.

## Results

Blood samples from radically cured individuals were genotyped for *msp2 *in order to distinguish new infections from recrudescent ones and to determine multiplicity of infection (MOI). All new infections with MOI = 1 or 2 were analysed by DNA Chip to identify SNPs in genes associated with drug resistance. In addition, genotyping on Chip was performed for all single and double infections found in baseline samples of these individuals prior to treatment. Complete haplotype data were obtained for 144 new infections and 109 baseline samples.

From cross sectional surveys, 61 samples with a complete haplotype were grouped as adults > 14 years and 63 samples derived from children aged five-14 years. Individuals that were treated with antimalarial drugs in the two months prior to the survey were excluded.

Genotyping was performed using DNA Chip technology [39]. Among the 25 analysed SNPs in the four different genes *Pfmdr*, *Pfcrt*, *Pfdhfr *and *Pfdhps *some occurred only as the wild type allele, whereas some mutant alleles had already reached fixation at this field site. For 8 SNPs both the wild type and mutant allele was detected in the samples set.

At this study site a total of 13 different haplotypes were found, all listed in table 1. The most frequent haplotype in all subsets of the host population was the 7-fold mutated haplotype '*crt*76T-*crt*356L-*crt*326D-*crt*220S-*dhfr*59R-*dhfr*108N-*mdr*86Y'. The wild type allele was fixed at the remaining 4 polymorphic codons.

**Table 1:** Frequency of haplotypes in different subsets of the host population

									**adults vs. children**			**adults vs. new infections**		
									
**Haplotype**^1^	**Adults cross section^2^(n = 61)**		**Children cross section^2^(n = 63)**		**TRS^3^baseline samples (n = 109)**		**TRS^3^new infections (n = 144)**		**OR**	***p*^4^**	**95% CI**	**OR**	***p*^4^**	**95% CI**
	
	n	%	n	%	n	%	n	%						
*crt*76 *crt*326 *crt*356	0	0	0	0	0	0	1	0.69	-	-	-	-	-	-
*crt*76 *crt*326 *crt*356 *crt*220	1	1.64	1	1.59	0	0	0	0	0.97	0.98	0.06 – 15.83	-	-	-
*crt*76 *crt*326 *crt*356 *dhfr*108 *dhfr*59	0	0	0	0	3	2.75	0	0	-	-	-	-	-	-
*crt*76 *crt*326 *crt*356 *crt*220 *dhps*540	0	0	1	1.59			0	0	-	-	-	-	-	-
***crt*76 *crt*326 *crt*356 *crt*220 *mdr*86**	4	6.56	2	3.17	0	0	1	0.69	0.47	0.39	0.08 – 2.65	**0.1**	**0.04**	0.01 – 0.91
*crt*76 *crt*326 *crt*356 *crt*220 *dhfr*108 *dhfr*59	3	4.92	0	0	4	3.67	1	0.69	-	-	-	0.14	0.09	0.01 – 1.33
*crt*76 *crt*326 *crt*356 *dhfr*108 *dhfr*59 *mdr*86	8	13.11	4	6.35	15	13.76	8	5.56	0.45	0.21	0.13 – 1.58	0.39	0.07	0.14 – 1.09
*crt*76 *crt*326 *crt*356 *crt*220 *dhfr*108 *mdr*86	1	1.64	2	3.17	2	1.83	2	1.39	1.97	0.59	0.17 – 22.27	0.85	0.89	0.75 – 9.5
*crt*76 *crt*326 *crt*356 *dhfr*108 *dhfr*59 *mdr*86 *dhps*540	0	0	0	0	0	0	1	0.69	-	-	-	-	-	-
***crt*76 *crt*326 *crt*356 *crt*220 *dhfr*108 *dhfr*59 *mdr*86**	40	65.57	53	84.13	85	77.98	126	87.5	**2.78**	**0.02**	1.18 – 6.56	**3.68**	**0.001**	1.78 – 7.57
*crt*76 *crt*326 *crt*356 *crt*220 *dhfr*108 *dhfr*59 *mdr*86 *dhps*540	2	3.28	0	0	0	0	4	2.78	-	-	-	0.84	0.85	0.15 – 4.73
*crt*76 *crt*326 *crt*356 *crt*220 *dhfr*108 *dhfr*59 *mdr*1042 *mdr*184	1	1.64	0	0	0	0	0	0	-	-	-	-	-	-
*crt*76 *crt*326 *crt*356 *crt*220 *dhfr*108 *dhfr*59 *mdr*86 *dhps*437	1	1.64	0	0	0	0	0	0	-	-	-	-	-	-

^1) ^Haplotypes represent 11 single nucleotide polymorphisms (SNPs) in 4 different genes. The remaining 14 SNPs tested but not listed equal to wildtype.

^2) ^Cross-sectional samples include only individuals that have not been treated with antimalarial drugs in the 2 months prior to the survey.

^3) ^TRS, treatment to reinfection study

^4) ^Bold indicates p < 0.05

In the samples set, a number of haplotypes occurred at very low frequency (one or two observations). The detection of rare haplotypes depends on sample size and presence or absence of these haplotypes in a population comparison is likely due to chance.

### Effect of host age on haplotype frequency

**Figure 1:**
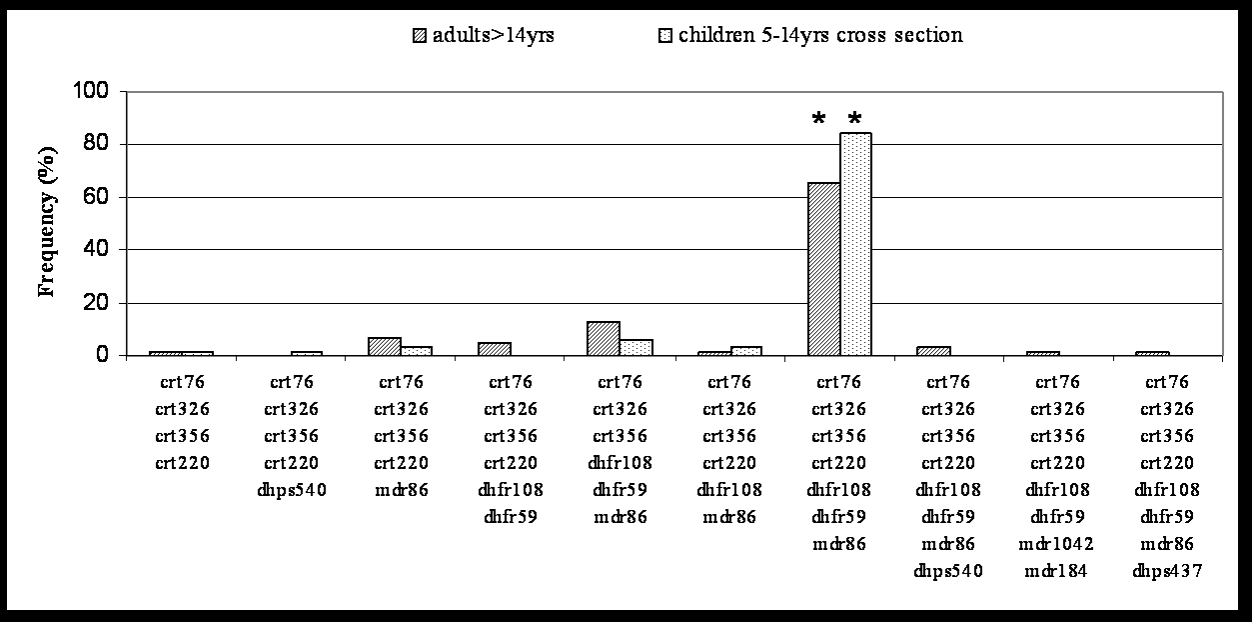
Comparison of haplotypes between adults and children of the cross-sectional surveys. * indicates significant difference of haplotype frequency between the two compared groups.

Haplotype frequencies in children and adults of the cross sectional surveys were compared in order to test for age-specific effects. The 7-fold mutated haplotype '*crt*76T-*crt*356L-*crt*326D-*crt*220S-*dhfr*59R-*dhfr*108N-*mdr*86Y' was the most frequent haplotype in adults and children with a prevalence of 65.57 % and 84.13 %, respectively (Table 1). The frequency of this haplotype was significantly lower in adults compared to children by more than 20% (OR = 2.78, p = 0.02) (Figure 1). In addition to the seven-fold mutated haplotype, seven additional haplotypes were found in adults, most of them occurring at very low frequency with the exception of the 6-fold mutant '*crt*76T-*crt*356L-*crt*326D-*dhfr*59R-*dhfr*108N-*mdr*86Y' which showed an increased frequency in adults compared to children (13.11% vs. 6.35%). This difference was not statistically significant due to the very few observations.

### Haplotypes in new infections

**Figure 2:**
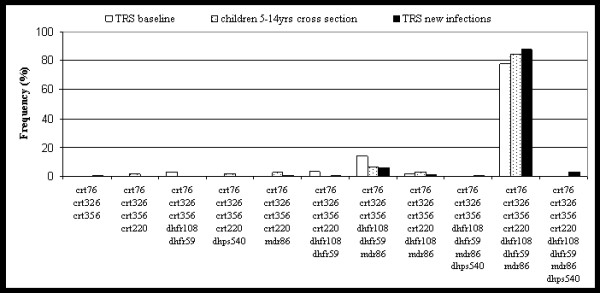
Comparison of haplotypes between children of the cross sectional surveys and baseline samples and new infections from the treatment to reinfection study (TRS). There are no significant differences in haplotype frequencies between groups.

Since new infections best reflect the haplotype frequencies that are actually transmitted, first infections after radical cure with artesunate monotherapy were genotyped. By genotyping the highly polymorphic *msp1 *and *msp2 *locus all recrudescent parasites due to treatment failure were excluded. Baseline samples prior to radical cure which had a single or double-clone infection were also genotyped. Figure 2 shows that haplotype frequencies agreed well in baseline samples and new infections, with the exception of some rare types which are probably fluctuating randomly. In addition, haplotype frequencies from age-matched cross sectional samples are indicated and also show good agreement with frequencies found in new infections. The fact that haplotypes in new infections appearing after radical cure, which likely reflect the transmitted parasite population, do not differ from haplotype frequencies in age matched children, suggests a high rate of clone acquisition in children. The concordance between baseline samples and samples from age-matched children proves that the data sets from the cross sectional survey and from the radical cure are comparable and unbiased. In all three subsets of the host population from Figure 2, the frequency of the dominant haplotype agreed well and showed no statistically significant difference.

### New infections versus persisting infections in adults

In order to determine whether all haplotypes persist in the host equally well, the haplotype distribution in newly arriving infections in relation to persistent parasites from long term infections were compared. Previously, it was shown by genotyping *P. falciparum *clones from a longitudinal study that individual parasite clones can persist over very long time in a semi immune host at very low densities fluctuating around the detection level [40,41]. To select long term persistent infections from this data set, infections from adults > 14 years were chosen (mean age = 31 years; SD = 13.67). To avoid any influence of previous drug intake on the prevalence of certain haplotypes, all patients treated with antimalarials two months prior to sampling were omitted from the analysis.

**Figure 3:**
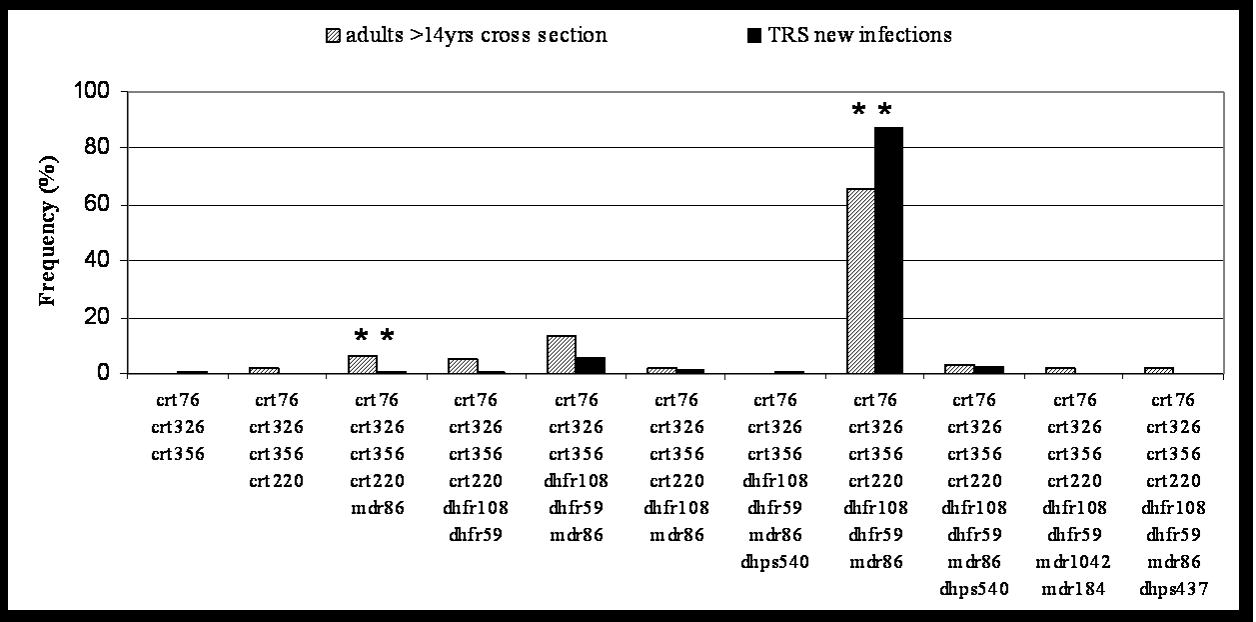
Comparison of haplotypes between adults (cross section) and new infections of the treatment to reinfection study. * indicates significant difference of haplotype frequency between the two compared groups.

Figure 3 compares new infections and persisting infections in adults. A significantly higher number of seven-fold mutated haplotype was found in new infections compared to frequencies in adults with a difference between the groups of 21.92% (OR = 3.68, p < 0.001; Table 1). In contrast, the frequency of the five-fold mutated haplotype '*crt*76T-*crt*356L-*crt*326D-*crt*220S-*mdr*86Y' was significantly lower in new infections (OR = 0.1, p = 0.04; Table 1). The frequency of 6-fold mutant '*crt*76T-*crt*356L-*crt*326D-*dhfr*59R-*dhfr*108N-*mdr*86Y' was also reduced in new infections, although this difference did not reach statistical significance. This suggests for adults a higher clearance rate for clones carrying seven-fold mutations and as a consequence, accumulation of five- and six-fold mutations.

## Discussion

The most frequent haplotype in the study area showed the following seven point mutations: *crt*76T *crt*326D *crt*356L *crt*220S *dhfr*59R *dhfr*108N *mdr*86Y. The high frequencies of these mutations were concordant with findings from previous studies in PNG [29,30]. Frequent mutations in *mdr1 *and *crt *reflect the long history of 4-aminoquinoline usage in the country and the high levels of resistance against these drugs [42]. High frequencies of *dhfr *mutations suggest that resistance to pyrimethamine is common and sulfadoxine, as indicated by the low level of mutations in *dhps*, is probably the only effective component in the locally used first line treatment combination of CQ or Amodiaquine with SP.

The study presented here demonstrated different haplotype frequencies in subsets of the host population. Children five to 14 years of age harboured more seven-fold mutated haplotypes compared to adults. The same frequency of this haplotype was observed in new infections as in children. Assuming that new infections, as they were observed after radical cure, reflect the actually transmitted haplotypes via the Anopheline vector, these findings suggest three interpretations: (i) transmission of highly mutated and therefore probably more resistant haplotypes is modulated by drug pressure or other environmental factors so far undetected in this study; (ii) transmission of drug resistance markers occurs in an age-dependent mode by which mutant parasites accumulate in children; or (iii), the parasite population found in children differs intrinsically from that in adults.

The first possible interpretation of the present findings implies that previous treatment effects the transmission of particular haplotypes. This is supported by the finding of higher prevalence and density of gametocytes following treatment with SP or CQ which was reported by a number of different authors [24-27], but is contradicted by a report from Dunyo *et al *[43] who could not find such an effect of SP treatment on subsequent transmission gametocyte carriage or density. In this study an altered transmission potential as a consequence to antimalarial treatment [44] was ruled out by excluding all individuals that had received antimalarial treatment two months prior to blood collection. Thus, effects of SP which is known to have a long elimination half life (4–9 days for Sulfadoxine and ca. 4 days for pyrimethamine) [45] should have waned. However, long lasting effects of SP treatment on transmission need to be further investigated.

An alternative explanation of the present findings implies that children and adults differ in their infectivity to mosquitoes. The age effect on transmission has been investigated by Graves and colleagues [46] in Madang, PNG, who performed direct mosquito feeding experiments on human blood. They found that the 1–20 year old individuals are more infectious to the mosquitoes than older age groups. However, a mathematical model developed by Ross *et al *[47] proposed that also infected adults are likely to make a substantial contribution to the infectious reservoir. The concordance of haplotype frequencies between new infections and children but not adults could suggest that children contribute more to transmission than adults in our study area. But such differential infectivity to mosquitoes is not the only explanation for the fact that frequencies in new infections do not differ from frequencies in age matched children. This could also be due to high turnover of infecting parasite clones in children. If in a particular age group clone acquisition rate is high, radically cured as well as non-cured individuals will all have predominantly recent infection and as a consequence will share the same haplotype frequencies of drug resistance markers.

As third possible explanation for heterogeneity in haplotype distribution other malariological parameters or host factors that have the potential to determine survival of the haplotypes in the host have to be considered. For example, *P. falciparum *infections in children differ from those in adults by a higher mean number of multiple infections [40,48,49] and higher parasite densities [49,50]. A significantly reduced parasite density has been associated with resistance patterns [51]. In the data presented here, a reduced parasite density in mutant samples compared to wild type could not be confirmed. Also the densities of the two most common haplotypes did not differ between adults and children (data not shown). As further determinant of parasite survival in the host, some authors have proposed that mutations associated with drug resistance will incur fitness costs to the parasite in absence of drug pressure [51-55]. Since parasite fitness cannot be measured directly, a surrogate marker for fitness is required. The parameter "persistence of a clonal infection", measured as duration of an infection in a given host, would serve this purpose and can be measured experimentally in a longitudinal set of samples. This leads to the speculation that long-term persistence of a clonal infection in a host indicates better survival and thus could be used as a surrogate marker for parasite fitness.

Such fitness costs of drug resistance mutations can obviously only be studied in the absence of treatment. The effect of previous treatment on infections of particular haplotypes was ruled out in this dataset by excluding all individuals that had received antimalarial treatment 2 months prior to blood collection.

A recent paper of Ord and colleagues [56] reported that the prevalence of two mutations associated with chloroquine resistance declined during the dry season. The authors suggest fitness costs of drug resistance to be responsible for reduced survival of mutant parasites. A similar seasonal fluctuation has been suggested for Sudan [57]. These findings from a longitudinal study in a seasonal setting are perfectly in line with our findings from an area of perennial transmission where a higher frequency of mutated haplotypes was detected in new infections as compared to long lasting chronic infections.

In malaria endemic areas where transmission is perennial, most adults carry asymptomatic infections. These infections largely remain untreated and reflect chronic infections that persist over long periods of time (about 150 days) [40]. In case of long-term survival within a host, less mutated parasites would be expected to be more frequent due to their higher fitness. The present observation of a reduced frequency of the seven-fold mutant in adults compared to new infections is in support of reduced fitness of this particular haplotype. The opposite is true for less mutated haplotypes with increased prevalence in adults who frequently carry chronic subpatent parasitaemia.

When looking at each SNP separately, it was found that the frequency of the four most prevalent mutant and non-fixed SNPs (*crt*220S, *dhfr*59R, *dhfr*108N and *mdr*86Y) was lower in chronic infections than in children or new infections. This also supports the hypothesis that infections harbouring fewer point mutations are fitter and can therefore persist in the absence of drug pressure. The frequency of other SNPs was very low and does not allow for any interpretation.

The host's acquired immunity is an important parameter that needs to be considered when using the persistence of infection as a measure of parasite fitness. Immunity is a major determinant of duration of infection. However, the hosts acquired immunity is unlikely linked with a specific drug resistance haplotype as lack of linkage disequilibrium and sufficient outbreeding has been shown previously for the parasite population in the study area [33]. Therefore the immune response is expected to act on parasites irrespective of their number of drug resistance mutations.

Fitness costs of drug resistance is one important parameter for mathematical models that remains to be quantified in order to make more precise predictions on the spread of drug resistance. If point mutations incur fitness costs to the parasite in the absence of drug pressure, natural selection might lead to a decline in the prevalence of these mutations once the use of a specific drug is abolished. This might then result in the drug becoming efficacious again as has been observed in Malawi in the 12 years since CQ was removed from standard treatment [58]. Longitudinal studies are needed in order to estimate and quantify the reduction in survival of mutated versus wildtype genotypes in the host. This could provide more precise fitness measurements for parasites harbouring point mutations that are associated with drug resistance.

## Authors' contributions

SS carried out the molecular genetic work and the statistical analysis. JM carried out the field survey and participated in the molecular genetic work. MG and MB carried out field work. IM was responsible for the treatment to reinfection study and participated in data analysis. IF was responsible for the study design and contributed to data analysis. All authors contributed to writing the manuscript.
